# Baseline Sarcopenia is Associated with Lack of Response to Therapy, Liver Decompensation and High Mortality in Hepatocellular Carcinoma Patients

**DOI:** 10.31557/APJCP.2020.21.11.3285

**Published:** 2020-11

**Authors:** Hanaa Badran, Maha Mohammad Elsabaawy, Amr Ragab, Rasha Abdelhafiz Aly, Ayman Alsebaey, Aliaa Sabry

**Affiliations:** 1 *Department of Hepatology and Gastroenterology, National Liver Institute, Menoufia University, Shebeen El-Koom, Egypt. *; 2 *Department of Diagnostic Medical Imaging and Interventional Radiology; National Liver Institute, Menoufia University, Shebeen El-Koom, Egypt. *

**Keywords:** Skeletal muscle index, sarcopenia, survival, hepatocellular carcinoma

## Abstract

**Objective:**

hepatocellular carcinoma (HCC) is a dreadful complication of liver cirrhosis. Aim was to study the effect of sarcopenia on the survival in patients with HCC.

**Methods:**

we included 262 patients and were followed up for 12 months. Sarcopenia was calculated by skeletal muscle index (SMI). Sarcopenia was defined by SMI ≤39 cm^2^/m^2^ for women and ≤50 cm^2^/m^2^ for men.

**Results:**

patients with sarcopenia (n= 113, 43.1%) were older, mainly males, Child-Pugh class B and smokers. Patients with sarcopenia had lower survival than those without (10.09 vs. 11.72 months). Survival was also lower in Barcelona clinic liver cancer stage C than B and A (9.02 vs. 11.21 vs. 11.89 months). Age and sarcopenia were hazardous of mortality (p<0.05). There was statistically significant difference of serial SMI in patients without baseline sarcopenia unlike patients with baseline sarcopenia. On follow up patients with sarcopenia had higher incidence of ascites (45% vs. 20.4%), spontaneous bacterial peritonitis (21.7% vs. 11.6%), hepatic encephalopathy (28% vs. 11.5%) and bleeding (22.9% vs. 12.7%). Totally patients with sarcopenia had higher incidence of progressive HCC (39% vs. 25.5%).

**Conclusion:**

Sarcopenia is associated with lack of response to therapy, liver decompensation and higher mortality in hepatocellular carcinoma patients.

## Introduction

Hepatocellular carcinoma (HCC) is a dreadful complication of the liver cirrhosis that is associated with short survival. In men, HCC is the fifth malignancy meanwhile in the women, it is the eighth malignancy (Elshaarawy et al., 2019).

The generalized loss of both the function and the mass of muscles, is called sarcopenia. Sarcopenia may be due to aging or secondary to medical disease like liver cirrhosis (Bunchorntavakul and Reddy, 2020). 

There are multiple factors that cause malnutrition and subsequent sarcopenia in patients with cirrhosis. Decreased nutrient intake is common owing to anorexia, protein restriction, disturbed conscious state and taste change. The energy metabolism is altered due to hypercatabolic state and altered metabolism for carbohydrates, proteins, and fat. Maldigestion is common. Another factor is repeated fasting with bleeding, encephalopathy and before procedures (Dasarathy and Merli, 2016; Bunchorntavakul and Reddy, 2020).

There are various methods for the diagnosis of sarcopenia such as the hand grip, anthropometry, bioelectrical impedance, dual-energy X-ray absorptiometry and computerized tomography (CT). In CT sarcopenia is diagnosed by measuring the skeletal muscle cross sectional at the third lumbar vertebra normalized to the height (Ebadi and Montano-Loza**)**.

Sarcopenia is an independent predictor of increased care cost, hepatic encephalopathy, waitlist mortality, longer hospital stay and post-liver transplantation mortality (Ebadi and Montano-Loza; Sinclair et al., 2016).

There are various consequences of malnutrition and sarcopenia in pateints with cirrhosis. There is increased incidence of hepatic encephalopathy and decreased quality of life. Bacterial complications are common with shortened survival. The decreased physical activity predisposes to peri or post-operative complications. HCC patients with sarcopenia suffer from increased treatment induced complications and decreased overall survival. Sarcopenia affects liver transplantation badly where there is increased waitlist mortality, peri/post-operative mortality with decreased survival (Dasarathy and Merli, 2016; Bunchorntavakul and Reddy, 2020). A recent review article mentioned the role of sarcopenia with all the modalities of HCC treatment and the effect on the survival (Marasco et al., 2020).

The aim of the study was to assess the effect of sarcopenia on the survival in patients with HCC.

## Materials and Methods

*Patients and Methods*

This study included 262 patients diagnosed to have HCC recently within 4 weeks without any management as regards the focal lesions. Hepatocellular carcinoma was diagnosed by triphasic CT in which the focal lesion showed arterial enhancement followed by portal and venous washout (European Association for the Study of the et al.). These patients were Barcelona clinic liver cancer (BCLC) stage A through C as patients with BCLC D were excluded from the beginning and those who were willing for liver transplantation.

All the patients underwent complete history taking and physical examination. The following investigations were done: liver function tests, kidney function tests, complete blood picture, INR and alfa-fetoprotein. The patients were followed up for 12 months.

Sarcopenia was diagnosed radiologically by the calculation of the skeletal muscle index (SMI). The SMI was defined as the cross-sectional area (cm^2^) of the muscles measured by CT at level of the third lumbar vertebrate that was normalized to the height of the patient. Sarcopenia was defined by SMI ≤39 cm^2^/m^2^ for women and ≤50 cm^2^/m^2^ for men (Carey et al., 2017).

Sarcopenia was classified morphologically using CT into 5 grades; 0 through 4 where grade 3 and 4 are advanced stages (Inokuchi et al., 2001). These morphological changes are the mean of the right and the left short to long axis of the psoas muscle (Inokuchi et al., 2001). 

Sarcopenia was assessed at inclusion (baseline) then after 1, 3, 6 and 12 months. At these time points patients were assessed also as regard the response to management and the occurrence of liver decompensation (ascites, hepatic encephalopathy, spontaneous bacterial peritonitis and upper gastrointestinal bleeding).

The management of the hepatocellular carcinoma was determined by the decision of the multidisciplinary HCC team which included hepatologist, radiologist and a hepatobiliary surgeon. The management included liver resection, radiofrequency ablation, microwave ablation, percutaneous ethanol injection, trans-arterial chemoembolization, oral sorafenib and the best supportive care.

This study was conducted in National Liver Institute hospitals, Menoufia University, Egypt. Patients were recruited from the outpatient hepatocellular carcinoma clinic. Approval from the institutional review board was obtained and the patients signed an informed signed consent before enrollment.

*Statistical Analysis*

Data were presented as mean ±standard deviation for normally distributed data and number (column percentage) for the categorical data. All p-values are 2 tailed where values <0.05 considered statistically significant. Comparisons between two groups were performed using the Student’s t-test for normally distributed. CHI-squared test (*χ*^2^) and Fisher exact test for categorical data analysis. Repeated measure ANOVA or Friedman test was used for testing serial changing variables. The survival as assessed by Kaplan-Meier survival. Comparison of survival curves was done by Logrank test. The Cox proportional-hazards regression was used to detect the predictors of mortality. Data were statistically analyzed using IBM^®^ SPSS^® ^Statistics^®^ version 21 for Windows. 

## Results

The comparison of the patients with and without sarcopenia is shown in Table 1. Patients with baseline sarcopenia (n= 113, 43.1%) were older, having lower body mass index and were being less categorized as BCLC A than non-sarcopenic patients. Sarcopenia was more common in the males compared to females. The presence of sarcopenia was not affected by the etiology of the liver disease. The CTP class B and smoking were common in patients with sarcopenia.

**Figure 1:**
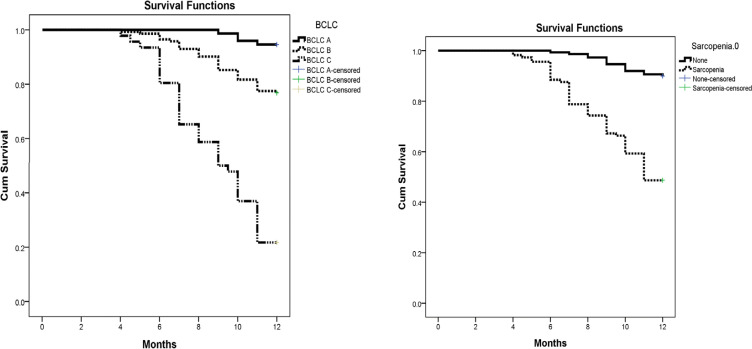
Kaplan-Meier Survival Analysis of the Effect of Sarcopenia and BCLC Staging

Patients with sarcopenia (Table 2 and Figure 1) had lower survival than those without (10.09 vs. 11.72 months). The survival was also lower in BCLC C than BCLC B and A (9.02 vs. 11.21 vs. 11.89 months).

The cox regression analysis revealed the following covariate that are associated with mortality (p<0.05); age (hazard ratio; 1.05, 95% CI: 1.01 - 1.08), BMI (hazard ratio; 0.71, 95% CI: 0.63 - 0.79), serum bilirubin (hazard ratio; 2.43, 95% CI: 1.66 - 3.54), serum albumin (hazard ratio; 0.25, 95% CI: 0.14 - 0.43), CTP B (hazard ratio; 4.73, 95% CI: 2.78 - 8.06), BCLC B (hazard ratio; 4.76, 95% CI: 1.68 - 13.42), BCLC C (hazard ratio; 23.98, 95% CI: 8.51 - 67.57) and sarcopenia (hazard ratio; 6.55, 95% CI: 3.71 - 11.58).

*Longitudinal follow up of patients with and without sarcopenia*

**Figure 2:**
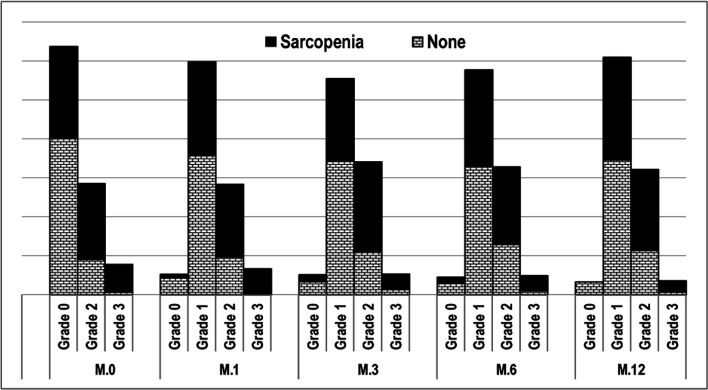
Grades of Sarcopenia in the Different Time Points of the Study

On follow up of patients with and without baseline sarcopenia after 1, 3, 6 and 12 months we found that the percentages of grade 2 and 3 sarcopenia were higher in patients with baseline sarcopenia, in these different time points (Table 1 and Figure 2).

**Figure 3:**
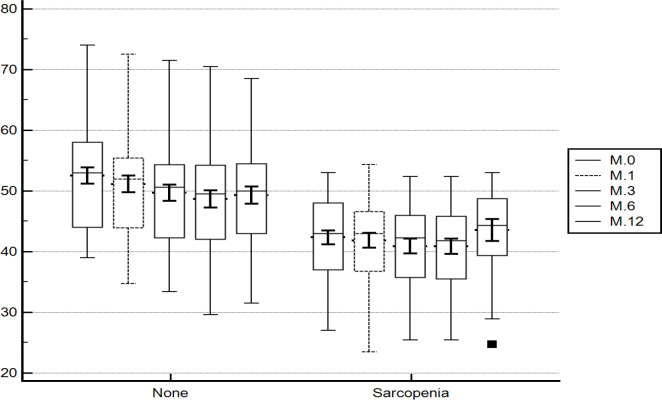
Tracing the Skeletal Muscle Index in the Different Time Points of the Study

On tracing the SMI (Figure 3) from the baseline through 1, 3, 6 and 12 months there was statistically significant difference (p =0.001) of the mean SMI in patients without baseline sarcopenia. The baseline value (cm^2^/m^2^) was statistically higher than the 1, 3, 6 and 12 months (52.78 ±8.32 vs. 51.80 ±8.41 vs. 50.34 ±8.12 vs. 49.54 ±8.28 vs. 49.28 ±8.29; p =0.001). On pairwise comparison of the different time points there was a statistically significant difference (p=0.001) between all of them except the value at 6 and 12 months which were comparable (p >0.005).

In patients with baseline sarcopenia, there was no statistically significant difference between the baseline and 1, 3, 6 and 12 months (44.12 ±5.34 vs. 43.85 ±5.77 vs. 43.90 ±5.45 vs. 43.52 ±5.31 vs. 45.72 ±13.23 cm^2^/m^2^; p =0.407).

On pairwise comparison of patients with and without sarcopenia in the different time points; there was a statistically significant difference (p =0.001) with higher values (cm^2^/m^2^) in patients without baseline sarcopenia (52.78 ±8.32 vs. 44.12 ±5.34), (51.80 ±8.41 vs. 43.85 ±5.77), (50.34 ±8.12 vs. 43.9 ±5.45), (49.54 ±8.28 vs. 43.52 ±5.31) and (49.28 ±8.29 vs. 45.72 ±13.23).

*Relationship between sarcopenia and the liver decompensation*

**Figure 4:**
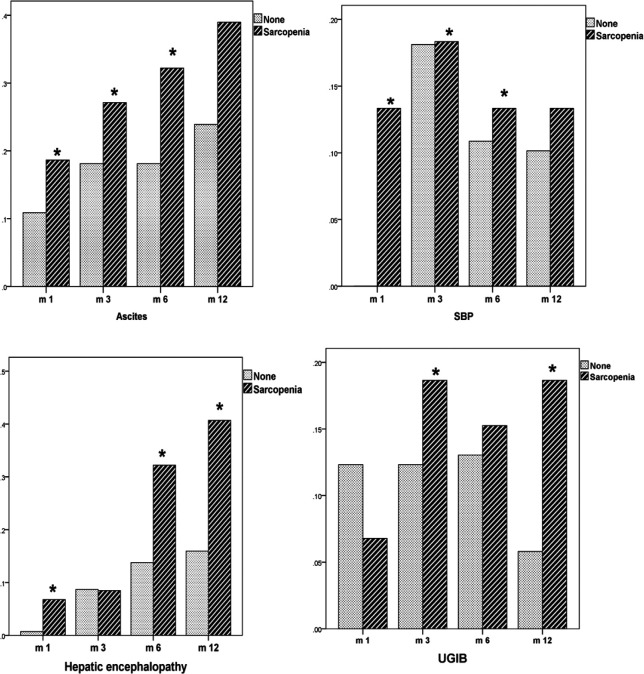
Liver Decompensation Incidence in the Different Time Ppoints of the Study

Patients with baseline sarcopenia had a statistically (p =0.01) higher percentage of ascites development in the first (34.5% vs. 12.1%), third (46% vs. 23.5%) and the sixth months (58.3% vs. 22.6%). On the end of the study as shown in Figure 4; the percentage was comparable (39% vs. 23.9%; p=0.32).

Patients with baseline sarcopenia had a statistically (p =0.01) higher percentage of spontaneous bacterial peritonitis in the first (10.9%vs. 0.7%), third (39.9% vs. 12.8%) and the sixth months (32.4% vs. 6.1%). On the end of the study the percentage was comparable (13.3% vs. 10.1%; p=0.512).

Patients with baseline sarcopenia had a statistically (p =0.01) higher percentage of hepatic encephalopathy in the first (13.3% vs. 1.3%), sixth (44.4% vs. 17.8%) and the 12^th^ months (40.7% vs. 15.9%). On the third months the percentage were comparable (20.4% vs. 11.4%; p =0.46)

Patients with baseline sarcopenia had a statistically (p =0.01) higher percentage of upper gastrointestinal bleeding in the third (30.1% vs. 14.1%) and the 12^th^ months (18.6% vs. 5.8%). The percentage was comparable in the first (16.8% vs. 14.1%; p=0.544) and the sixth month (24.1% vs. 16.4%; p=0.130).

Totally patients with sarcopenia compared to those without had higher (p =0.001) percentage of ascites (45% vs. 20.4%), spontaneous bacterial peritonitis (21.7% vs. 11.6%), upper gastrointestinal bleeding (22.9% vs. 12.7%) and hepatic encephalopathy (28% vs. 11.5%).

*Relationship between sarcopenia and response to treatment*

Patients with baseline sarcopenia compared to those without, had higher percentage of progressive HCC despite intervention in the first (29.2 vs. 16.1; p=0.023), third (40.7 vs. 29.5; p=0.001), sixth (51.4 vs. 30.6; p=0.001) and the 12th months (32.1 vs. 25.7; p=0.001).

Totally patients with sarcopenia compared to those without had higher (p =0.001) total percentage of progressive HCC (39% vs. 25.5%).

**Table 1:** Comparison of Patients with and without Sarcopenia

		Sarcopenia		Total	P-value
		None	Sarcopenia		
Age years (M±SD)		58.43 ±7.73	61.18 ±8.32	59.61 ±8.09	0.006
BMI k/m^2^ (M±SD)		25.17 ±2.4	23.20 ±2.06	24.32 ±2.46	0.001
BCLC N (%)	BCLC A	56 (37.6)	18 (15.9)	74 (28.2)	0.001
	BCLC B	79 (53)	63 (55.8)	142 (54.2)	
	BCLC C	14 (9.4)	32 (28.3)	46 (17.6)	
Etiology N (%)	Viral negative	3 (2)	3 (2.7)	6 (2.3)	0.909
	HBV	9 (6)	8 (7.1)	17 (6.5)	
	HCV	133 (89.3)	100 (88.5)	233 (88.9)	
	NASH	3 (2)	2 (1.8)	5 (1.9)	
	Others	1 (0.7)	0 (0)	1 (0.4)	
Sex N (%)	Female	53 (35.6)	27 (23.9)	80 (30.5)	0.042
	Male	96 (64.4)	86 (76.1)	182 (69.5)	
Smoking N (%)	Nonsmoker	123 (82.6)	77 (68.1)	200 (76.3)	0.024
	Smoker	11 (7.4)	14 (12.4)	25 (9.5)	
	Exsmoker	15 (10.1)	22 (19.5)	37 (14.1)	
Child-Pugh N (%)	A	96 (64.4)	50 (44.2)	146 (55.7)	0.001
	B	53 (35.6)	63 (55.8)	116 (44.3)	
Sarcopenia baseline N (%)	Grade 0	120 (80.5)	53 (46.9)	173 (66)	0.001
	Grade 2	27 (18.1)	44 (38.9)	71 (27.1)	
	Grade 3	2 (1.3)	16 (14.2)	18 (6.9)	
Sarcopenia 3^th ^month N (%)	Grade 0	10 (6.7)	4 (3.5)	14 (5.3)	0.001
	Grade 1	102 (68.5)	48 (42.5)	150 (57.3)	
	Grade 2	33 (22.1)	52 (46)	85 (32.4)	
	Grade 3	4 (2.7)	9 (8)	13 (5)	
Sarcopenia 6^th^ month N (%)	Grade 0	9 (6.1)	3 (2.9)	12 (4.8)	0.006
	Grade 1	97 (66)	51 (49.5)	148 (59.2)	
	Grade 2	38 (25.9)	41 (39.8)	79 (31.6)	
	Grade 3	3 (2)	8 (7.8)	11 (4.4)	
Sarcopenia 12^th^ month N (%)	Grade 0	9 (6.6)	0 (0)	9 (4.8)	0.006
	Grade 1	94 (69.1)	28 (52.8)	122 (64.6)	
	Grade 2	31 (22.8)	22 (41.5)	53 (28)	
	Grade 3	2 (1.5)	3 (5.7)	5 (2.6)	

**Table 2:** Kaplan-Meier Survival Analysis to Assess the Effect of the BCLC Score and the Sarcopenia

	Mean	95% C.I	P-value
BCLC			
BCLC A	11.89	11.78 - 12	0.001
BCLC B	11.21	10.93 - 11.5	
BCLC C	9.02	8.32 - 9.72	
Overall	11.02	10.79 - 11.25	
Sarcopenia			
None	11.72	11.57 - 11.88	0.001
Sarcopenia	10.09	9.65 - 10.53	
Overall	11.02	10.79 - 11.25	

## Discussion

HCC is an ominous complication of liver cirrhosis. It is associated with high mortality (Marasco et al., 2020). Recently sarcopenia was found to be a risk factor for decreased the overall survival in HCC patients (Levolger et al., 2015; Begini et al., 2017; Imai et al., 2017). 

In patients who underwent trans-arterial chemoembolization; the presence of baseline sarcopenia was associated with decreased overall survival (Loosen et al., 2019) unlike other studies who did not found difference (Kobayashi et al., 2018; Fujita et al., 2019). A recent study reported that the presence of sarcopenia is associated with high mortality and does not affect locoregional therapy safety (Lanza et al., 2020).

Harimoto et al., (2013) reported that patients who underwent resection with pre-operative sarcopenia had statistically lower survival and recurrence-free survival. This was confirmed by other studies (Voron et al., 2015; Harimoto et al., 2016; Takagi et al., 2016; Hiraoka et al., 2018; Kobayashi et al., 2019).

Sarcopenia is associated with higher incidence of recurrence following surgical resection and radiofrequency ablation of HCC (Kamachi et al., 2016).

In the different studies by Hiraoka et al.,(2017), Nishikawa et al., (2017) and Takada et al.,(2018) conducted on sorafenib treatment in HCC patients; about ~60% of the patients had sarcopenia. The overall survival was significantly less in patients with pretreatment sarcopenia.

AFP >100 mg/dL is a predictor of sarcopenia in HCC patients who are prepared for liver transplantation (Acosta et al., 2019). In the patients who were transplanted beyond the Milan, the HCC recurrence was higher in patients with pretransplant sarcopenia (Kim et al., 2018).

In our study, patients with sarcopenia were older and having advanced BCLC stage. The overall survival was less in patients with baseline sarcopenia in accord with other studies (Levolger et al., 2015; Begini et al., 2017; Imai et al., 2017) and in contrast to other studies (Kobayashi et al., 2018; Fujita et al., 2019). Moreover, the survival was higher in BCLC A patients. 

Patients with baseline sarcopenia had higher incidence of liver decompensation e.g. ascites, spontaneous bacterial peritonitis, hepatic encephalopathy, upper gastrointestinal bleeding during the study.

Furthermore, the baseline sarcopenia was associated with lack of response to intervention and progressive HCC.

On tracing of the skeletal muscle index, we found comparable values at the different time points of the study for patient with baseline sarcopenia. In contrast, the skeletal muscle index was decreasing from the baseline in patients without baseline sarcopenia. It may be due to the fact that most treatments are not curative till now for HCC.

In conclusion, Sarcopenia is associated with lack of response to therapy, liver decompensation and higher mortality in hepatocellular carcinoma patients.
